# QL1604 combined with bevacizumab as an innovative first-line treatment for HCC patient with extensive metastasis who showed remarkable effect: a case report

**DOI:** 10.3389/fphar.2024.1364871

**Published:** 2024-05-20

**Authors:** Yanzhi Wan, Keqin Tan, Hong Zhu

**Affiliations:** Department of Medical Oncology, Cancer Center, West China Hospital, Sichuan University, Chengdu, China

**Keywords:** hepatocellular carcinoma, QL1604, bevacizumab, intraspinal metastasis, paralysis

## Abstract

Hepatocellular carcinoma (HCC) is a common and highly malignant tumor with poor outcomes, especially when it metastasizes. In this report, we present the case of a 64-year-old male patient diagnosed with recurrence and multiple metastases of HCC 7 years after surgery. As the tumor invaded the spinal canal and pressed on the spinal cord, the patient experienced paralysis in the lower limbs. After undergoing surgical resection for spinal decompression, the patient chose an innovative regimen: QL1604 200 mg every 3 weeks plus bevacizumab 675 mg every 3 weeks as first-line treatment. From July 2022 to February 2024, the patient has regularly received the treatment. During the treatment, the paralysis symptoms of the patient gradually improved, and the motor function of the lower limbs completely returned to normal. When re-evaluated his spinal cord injury, the Frankel grade of the patient was downgraded from C to E. The tumor shrank to reach a state of PR and lasted for one and a half years. QL1604 combined with bevacizumab demonstrated excellent efficacy and minimal side effects in this patient. This new combined therapy holds potential as a first-line treatment strategy.

## Introduction

Hepatocellular carcinoma (HCC) is the most common primary tumor of the liver and has a very poor prognosis (Nagaraju et al., 2022). In China, HCC incidence is higher due to the prevalence of hepatitis B infection among the majority of patients, resulting in a greater malignant potential of HCC (Tanaka et al., 2011). HCC is known for its tendency to develop portal tumor thrombosis, intrahepatic metastasis, and lung metastasis. However, bone metastasis is rare in HCC (Bhatia et al., 2017).

For patients with advanced HCC, comprehensive treatment is crucial. Available options include systemic treatments, such as immunotherapy and targeted therapy, and local treatments, such as transarterial chemoembolization, hepatic artery infusion chemotherapy, radiotherapy, and so on (Llovet et al., 2021). Sorafenib and lenvatinib are the standard first-line single-agent targeted therapies for advanced HCC. In recent years, there have been breakthroughs in HCC treatment, particularly in systemic therapies evolving from single-agent targeted therapy to the combination of immunotherapy and targeted therapy (Yang et al., 2023). Nevertheless, immunotherapy combined with bevacizumab as the standard first-line treatment for HCC has shown better efficacy and fewer side effects (Ren et al., 2021).

In the case presented here, a patient with HCC developed intraspinal metastasis, leading to compression of the spinal cord and subsequent lower limb paralysis. Following emergency surgical resection for metastasis and spinal decompression, the patient chose QL1604 plus bevacizumab as the first-line treatment. QL1604 is a highly selective, humanized anti-PD-1 monoclonal antibody (mAb). It remains an experimental drug that has been studied in at least three clinical trials for solid tumors (Huang et al., 2023). Surprisingly, after using this treatment strategy, the patient achieved a state of partial response (PR) and has maintained this response for one and a half years. Throughout the treatment, adverse reactions were minimal, and the paralysis was completely cured, enabling the patient to move and resume normal daily life activities. QL1604 combined with bevacizumab exhibited significant efficacy in this patient. This case provides a new treatment plan for advanced liver cancer patients, hoping that more patients can benefit from it.

## Case presentation

### Pre-treatment

A 64-year-old male patient diagnosed with chronic hepatitis B infection for over 30 years ago underwent a radical complex liver cancer resection in 2015. Following the surgery, the patient opted for intermittent use of oral traditional Chinese medicine instead of any antitumor therapy or regular follow-up. Unfortunately, in January 2022 (7 years after the surgery), the patient began experiencing lumbosacral pain, which progressively worsened. Symptoms included numbness in the lumbosacral and left hip area, difficulty in walking, and paralysis. In March 2022, the patient visited our hospital for a thorough diagnosis and subsequent treatment.

**FIGURE 1:**
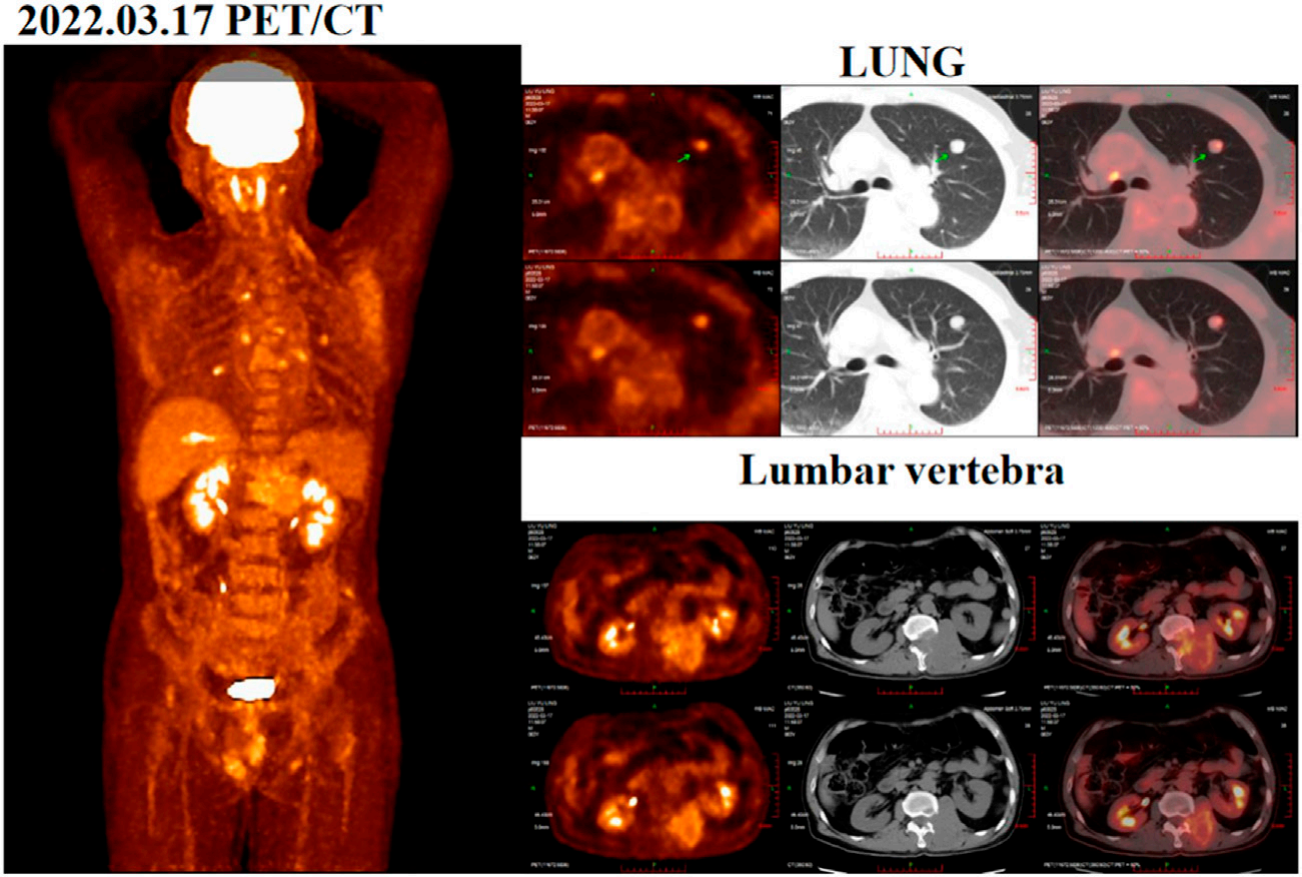
Positron emission tomography/computed tomography (PET/CT) images acquired before surgery on 17 March 2022.

During the physical examination, the patient was found to have decreased skin sensation below the left groin area, reduced tendon reflexes in both knees, and decreased muscle strength in both lower limbs. He was classified with “incomplete” injuries (grades C) using the Frankel system. A positron emission tomography/computed tomography (PET/CT) scan conducted in March 2022 revealed an increase in residual liver margin glucose metabolism following the liver cancer surgery. Additionally, abnormal glucose metabolism was observed in a nodule in their left upper lung, first lumbar vertebrae, and left adnexa (Figure 1). A chest-enhanced computed tomography (CT) scan showed a 1.3 × 1.5 cm lesion in the left upper lung, suggesting possible recurrence and metastasis of HCC. Besides, his liver function was Child-Pugh grade A and ECOG performance status score was 2. As part of the diagnostic process and to alleviate symptoms, the patient underwent surgery (lumbar-tumor excision plus spinal decompression) on 1 April 2022. Intraoperatively, extensive osteolytic destruction involving the lumbar and vertebral bodies was observed. Preoperative and postoperative digital radiographs of the thoracolumbar vertebrae were obtained, and the postoperative pathological examination indicated metastatic carcinoma in the first lumbar vertebrae. The results of immunohistochemistry staining of lumbar biopsy tissue were: CK8/18 (+), Hepar (+), GATA-3 (−), EMA (−), CK7 (−), CDX-2 (−), PAX-8 (−), GPC-3 (+), AFP (−), confirming the metastasis of HCC. Starting from postoperative, patients regularly use bisphosphonates and wear lumbar spine stents for a long time to reduce the risk of bone adverse events.

### In treatment

**FIGURE 2:**
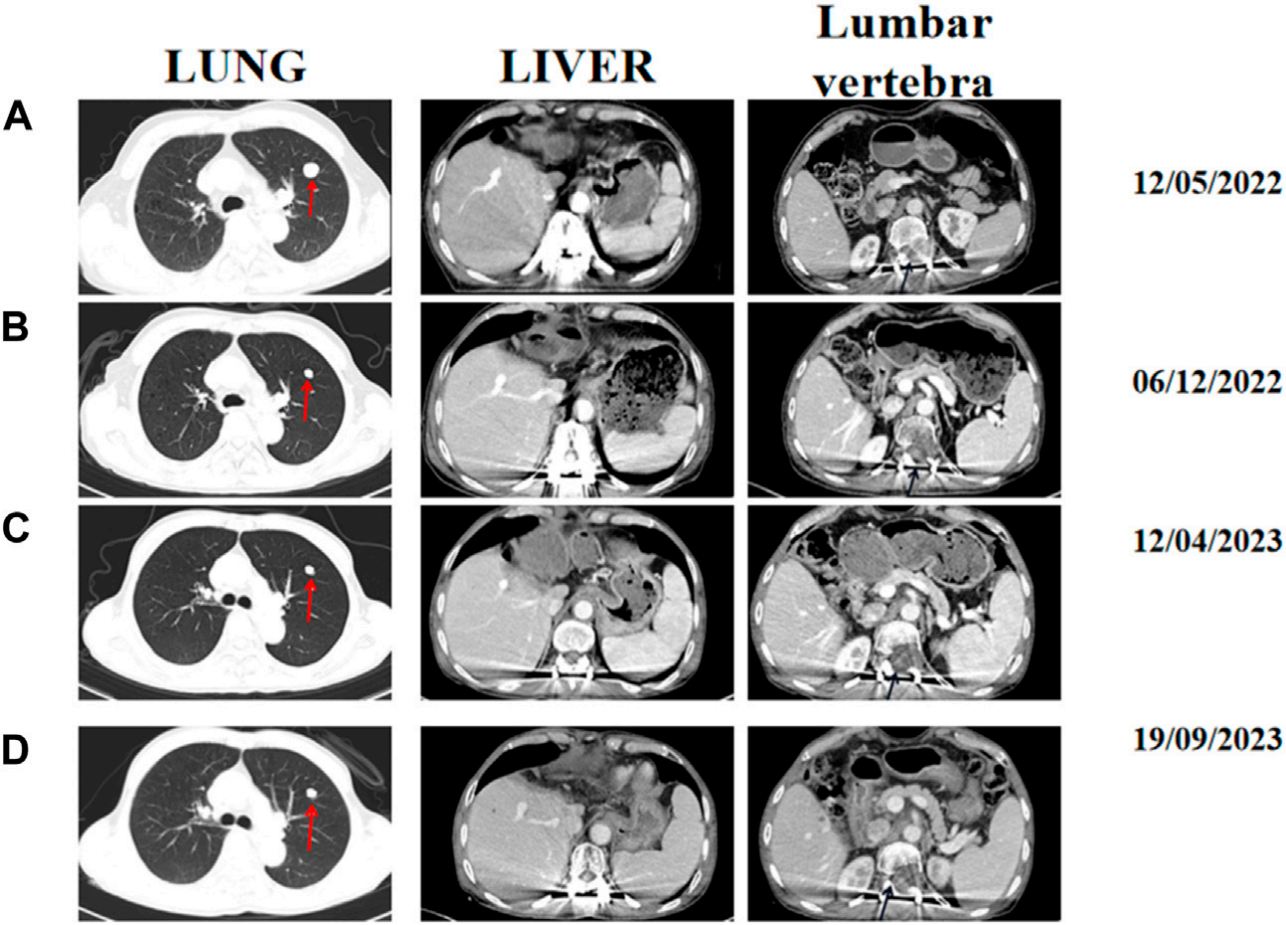
Representative abdominal and chest-enhanced CT images acquired at different times during treatment. **(A)** Baseline CT images acquired on 12 May 2022. **(B)** CT image assessment performed on 6 December 2022. **(C)** CT image assessment performed on 12 April 2023. **(D)** CT images acquired on 19 September 2023. The red arrows indicate lung lesions, and black arrows indicate lumbar soft tissue.

Two months post-surgery, following thorough discussions with the patient, it was mutually agreed upon to enroll the patient in a phase Ib/II clinical study to evaluates the safety, pharmacokinetics, and initial efficacy of QL1706/QL1604 in combination with bevacizumab for advanced HCC. Prior to enrollment, the patient underwent comprehensive assessment of their condition. The patient was diagnosed with stage C HCC of the Barcelona Clinic Liver Cancer with Child-Pugh A and an ECOG performance status score of 1. A baseline contrast-enhanced CT conducted in May 2022 confirmed a 1.7 × 1.5 cm reinforced nodule with lobed margins in the upper lobe of the left lung, along with scattered multiple lesions measuring 0.2–0.3 cm in both the lungs. Partial absence of the liver with a lamellar residual margin, slightly low density, and a slightly enhanced shadow were also observed. Furthermore, bone disruption was noted in the 12th thoracic spine, first lumbar vertebra, and left adnexa (Figure 2A). From July 2022 onward, the patient has received regular treatment with QL1604 (200 mg) every 3 weeks combined with bevacizumab (675 mg) every 3 weeks. During the treatment, the patient underwent chest and abdominal enhanced CT multiple times to reexamine their condition. Each time, the efficacy of the treatment was assessed consistently as a PR (Figures 2B, C).

### Post-treatment

The most gratifying thing is that the patient’s quality of life has significantly improved. Until now, the paralysis symptoms have been completely resolved, and the patient had experienced minimal side effects. After treatment, the lung metastasis decreased in size and the bone destruction of the affected vertebrae had also diminished (Figure 2D). There was a significant decrease in tumor markers such as serum alpha fetoprotein (AFP) and protein induced by vitamin K deficiency/antagonist II (PIVKA-II) of the patient. Additionally, the patient’s clinical symptoms improved significantly, and his Frankel grade was downgraded from C to E, indicating that sensory and motor functions below the injury plane have almost completely recovered. Furthermore, the patient’s ECOG performance status score decreased from 1 to 0, reflecting a substantial improvement in his overall health and functionality. Remarkably, the patient has survived for over 2 year despite multiple systemic metastases and have achieved a positive therapeutic outcome without experiencing any serious treatment-related adverse reactions. Until February 2024, the lung metastasis increased in size, however, the patient is still maintain a good sensory and motor function until now.

## Discussion

HCC is the most common primary malignancy of the liver, with a poor prognosis and a high rate of recurrence and metastasis (Nagaraju et al., 2022). Even with curative-intent options, the probability of recurrence and metastasis in patients with HCC within 5 years remains as high as 70% (Akateh et al., 2019). Moreover, epidemiological statistics show that in most of Asia and Africa, 60% of HCC cases are associated with a long history of HBV infection (Iavarone and Colombo, 2013). In the present case, our patient developed HCC due to a long history of chronic hepatitis without antiviral therapy. Unfortunately, it is worth noting that patients with liver cancer and hepatitis B virus infection often have a worse prognosis.

The patient initially experienced lumbosacral pain, followed by paralysis and was diagnosed with HCC recurrence and metastasis 7 years after surgery. While extra-hepatic metastasis in HCC often affects the lungs, bone metastases are relatively rare (Bhatia et al., 2017). In particular, metastatic epidural spinal cord compression (MESCC) is a devastating complication of cancer, usually caused by the metastasis of soft tissue to the epidural space. It is considered a medical emergency, and if the patient is nonambulatory or paraplegic, the chances of functional recovery are significantly reduced. Treatment options for MESCC include corticosteroids, radiotherapy, and surgery (Grant et al., 1994; Cole and Patchell, 2008). In the present case, due to the pressure of the tumor, the patient’s symptoms progressed rapidly. Upon visiting the hospital, the spinal cord compression symptoms were so severe that the patient had lost the ability to move and was bedridden. Therefore, the patient had to undergo an emergency surgery (lumbar tumor excision plus spinal decompression) in April 2022. The postoperative pathological result confirmed the metastasis of HCC. Studies have shown that if MESCC progresses slowly, decompression, even when performed 7 days after the initial onset, can reverse neurological deficits (Rades and Abrahm, 2010). However, despite undergoing surgery, the patient experienced compression symptoms for over a month and only achieved partial relief of pain and neurological recovery.

Despite the breakthrough in the treatment of HCC in recent years, the median survival time of HCC combined with bone metastasis (BM) is less than half a year (Guo et al., 2019). Unfortunately, The patient was found to have both bone and lung metastases, which worsened his prognosis. Radiotherapy and surgical treatment aim to alleviate pain symptoms in patients, but cannot improve bone destruction caused by bone metastasis (Kodama et al., 2007). The patient requires systemic anti-tumor treatment.

In recent years, we entered the immune era of liver cancer treatment. Immunotherapy combined with targeted therapy has become a new research focus (Yang et al., 2023). Combination treatment regimens for the first-line treatment of HCC, including the use of atezolizumab + bevacizumab and durvalumab + tremelimumab have been approved by FDA (Kudo, 2022; Yang et al., 2023). In our country, a phase 2-3 study (ORIENT-32) showed that in the first-line setting for Chinese patients with unresectable HCC, there was a significant effect of sintilimab (a PD-1 inhibitor) combined with bevacizumab compared with sorafenib (Ren et al., 2021). Immunotherapy combined with bevacizumab has become a standard first line treatment for HCC which has shown better efficacy and fewer side effects than a single target drug (Ren et al., 2021). Previous studies have shown that the etiology of HCC impacts the immune response and leads to unique microenvironmental features. However, the microenvironment of HBV-related HCC is more immunosuppressive and depleted than that of non-viral-related HCC. Therefore, HCC caused by chronic viral hepatitis B may be more suitable for immunotherapy (Lim et al., 2019; Llovet et al., 2022; Liu et al., 2023). Furthermore, incorporating antiangiogenic drugs into immune checkpoint inhibitors (PD-L1/PD-1) can enhance the antitumor immune response by synergistically regulating the tumor vasculature and immune microenvironment (Xing et al., 2021).

After extensive communication with the patient, the decision was made to opt for QL-1604 (a PD-1 inhibitor) combined with bevacizumab as the first-line treatment. The patient chose this treatment plan primarily because anti-PD-1 immune checkpoint inhibitor combined with bevacizumab is considered as a standard treatment option in China (Ren et al., 2021), and also due to the poor economic conditions of the patient’s family. The patient hoped to treat the disease by participating in a free clinical trial. Bevacizumab, a monoclonal antibody, targets vascular endothelial growth factor and inhibits angiogenesis and tumor growth. Notably, this medication stands as one of the first targeted therapies and the first approved angiogenesis inhibitor.

In contrast, QL1604, an anti-PD-1 immune checkpoint inhibitor, is a novel and unmarketed inhibitor. The clinical trial protocol involved the regular use of the immunotherapy QL1604 (anti-PD-1) in combination with bevacizumab, along with scheduled assessments. This new clinical trial aimed to further explore the efficacy of immunotherapy in patients with advanced HCC. It is important to note that this strategy is not a standard first-line therapy, and no previous research has shown that QL1604 combined with bevacizumab alone is an effective treatment for a patient with advanced HCC.

A clinical study IMbrave150 has shown that the median progression free survival (PFS) was 6.8 months in advanced HCC patients treated with atezolizumab plus bevacizumab (Finn et al., 2020). According to ORIENT-32, a clinical study in China, the median PFS of patients treated with combination therapy of sintilimab and bevacizumab was 4.6 months (Ren et al., 2021). A Phase 3 HIMALAYA trial showed a median PFS of 3.8 months in advanced HCC patients treated with Durvalumab combined with Tremelimumab (Kudo, 2022). Surprisingly, the PFS of the patient who treated with QL1604 + bevacizumab reached one and a half years. Even better, the patient is now completely cured of his paralysis. The patient is able to move freely, exceeding all expectations. The synergistic effect of the combined drug regimen surpasses that of individual treatments alone, highlighting the potential of such combination therapies in HCC. This case inspires us to conduct further investigations into the combination treatment regimen for liver cancer and reaffirms the significant role of immunotherapy alongside antivascular drugs in managing HCC.

In this particular case, two challenging events occurred during treatment. Specifically, before treatment, the patient developed intraspinal metastasis, a series of symptoms of spinal cord compression, and paralysis. Notably, few reports have focused on the successful first-line treatment of such an advanced case of HCC with QL1604 (a new PD-1 inhibitor) plus bevacizumab. There is still relatively little information on the efficacy of immunotherapy for bone metastases (Lasagna et al., 2021). The patient had demonstrated a state of PR for one and a half years, resulting in a notable improvement in the quality of life. Furthermore, the side effects of the treatment were observed to be mild and tolerable.

## Conclusion

With the continuous advancement of biological knowledge, more and more new drugs have begun to be applied. Additionally, the strategy of immunotherapy combined with targeted therapy has been increasingly recognized in HCC treatment. In this case, a patient with HCC in both lung and intraspinal metastasis experienced progression-free survival for one and a half years after systemic therapy with QL1604 and bevacizumab. Its efficacy was evaluated based on the patient’s sustained PR and complete recovery from paralysis. Accordingly, the combination of QL1604 and bevacizumab may be a better first-line treatment than the individual drugs alone for HCC. However, further prospective studies are required to confirm this hypothesis.

## Data Availability

The original contributions presented in the study are included in the article/supplementary material, further inquiries can be directed to the corresponding author.
